# K-RAS Mutant Pancreatic Tumors Show Higher Sensitivity to MEK than to PI3K Inhibition *In Vivo*

**DOI:** 10.1371/journal.pone.0044146

**Published:** 2012-08-31

**Authors:** Irmgard Hofmann, Andreas Weiss, Gaelle Elain, Maria Schwaederle, Dario Sterker, Vincent Romanet, Tobias Schmelzle, Albert Lai, Saskia M. Brachmann, Mohamed Bentires-Alj, Thomas M. Roberts, William R. Sellers, Francesco Hofmann, Sauveur-Michel Maira

**Affiliations:** 1 Novartis Institutes for Biomedical Research, Oncology Disease Area, Basel, Switzerland; 2 Novartis Institutes for Biomedical Research, Oncology Disease Area, Emeryville, California, United States of America; 3 Friedrich Miescher Institute for Biomedical Research, Basel, Switzerland; 4 Dana Farber Cancer Institute, Boston, Massachusetts, United States of America; 5 Novartis Institutes for Biomedical Research, Oncology Disease Area, Cambridge, Massachusetts, United States of America; Enzo Life Sciences, Inc., United States of America

## Abstract

Activating K-RAS mutations occur at a frequency of 90% in pancreatic cancer, and to date no therapies exist targeting this oncogene. K-RAS signals via downstream effector pathways such as the MAPK and the PI3K signaling pathways, and much effort has been focused on developing drugs targeting components of these pathways. To better understand the requirements for K-RAS and its downstream signaling pathways MAPK and PI3K in pancreatic tumor maintenance, we established an inducible K-RAS knock down system that allowed us to ablate K-RAS in established tumors. Knock down of K-RAS resulted in impaired tumor growth in all pancreatic xenograft models tested, demonstrating that K-RAS expression is indeed required for tumor maintenance of K-RAS mutant pancreatic tumors. We further examined signaling downstream of K-RAS, and detected a robust reduction of pERK levels upon K-RAS knock down. In contrast, no effect on pAKT levels could be observed due to almost undetectable basal expression levels. To investigate the requirement of the MAPK and the PI3K pathways on tumor maintenance, three selected pancreatic xenograft models were tested for their response to MEK or PI3K inhibition. Tumors of all three models regressed upon MEK inhibition, but showed less pronounced response to PI3K inhibition. The effect of MEK inhibition on pancreatic xenografts could be enhanced further by combined application of a PI3K inhibitor. These data provide further rationale for testing combinations of MEK and PI3K inhibitors in clinical trials comprising a patient population with pancreatic cancer harboring mutations in K-RAS.

## Introduction

The small GTPase K-RAS is frequently mutated in human cancers, with mutations occurring in 90% of non neuro-endocrine pancreatic tumors [1]. The presence of these mutations locks the protein in a constitutively activated form, which in turn results in enhanced stimulation of proliferative pathways, thus conferring a growth advantage on the cancer cell [2]. A number of genetic studies have shown that such activating K-RAS mutations are necessary for the onset of pancreatic cancer [3]–[5]. An inducible pancreas-specific expression system was used recently to show that K-RAS^G12D^ expression is also required for tumor maintenance [6]. This renders K-RAS a highly validated target for which specific inhibitors are expected to lead to antitumor efficacy. Unfortunately, all attempts to develop such molecular entities have failed so far, placing this target in the so-called difficult-to-drug target category [7]–[8]. Alternative strategies rely on inhibition of key downstream effectors, an approach reminiscent to the hunt for synthetic lethal interactors [9].

K-RAS signals via a number of downstream effectors, amongst others RAF kinase, PI3 kinase (PI3K), exchange factors for the small GTPases RAL and RAC as well as phospholipase C ε [10]. The RAF-MEK-ERK (MAPK) and the PI3K pathways are well described mediators of RAS induced transformation and tumorigenesis [11]–[12]. The *in vivo* significance of PI3K in K-RAS mediated tumorigenesis in the lung has been demonstrated using mice genetically engineered to carry a PI3K mutation deficient in RAS binding [13]. However, the role of either pathway in tumor maintenance is less clear. In the lung, it appears that MAPK signaling plays a more important role in tumor maintenance than PI3K signaling, since treatment of established K-RAS mutant lung tumors was more effective using MEK inhibitors than using PI3K inhibitors [14]–[15]. In pancreatic tumors, there are hints that the PI3K as well as the MAPK pathway might be involved in tumor maintenance [16]–[19]. However, the function of these pathways in tumor maintenance of the pancreatic lineage still needs further elucidation, since a better understanding of the contribution of K-RAS effectors to tumor maintenance might help to identify therapies alternative to targeting K-RAS itself.

There is a trend towards treatment with combinations of inhibitors rather than with single inhibitors. The importance of tumor-host interactions is well known in the case of pancreatic cancer, with hedgehog as well as PI3K signaling playing an important role in regulating the tumor stroma [20]–[21]. Targeting both tumor cells as well as the tumor stroma might therefore be necessary to effectively treat such cancers. Furthermore, in K-RAS mutant tumors in which K-RAS signals via multiple effector pathways, inhibition of several of these pathways is likely to be more effective than targeting just a single one. Finally, there are feedback loops between the MAPK and the PI3K pathway, which can result in activation of one pathway upon inhibition of the other, and in this way confer resistance to single agent treatment [15], [22]–[23]. Combinations of MEK and PI3K inhibitors have been tested in models of K-RAS mutant breast, lung and colorectal cancer, and were shown to be superior to single agent treatment [14]–[15], [24]–[26]. It remains to be seen if such combination treatment can be successfully applied to K-RAS mutant pancreatic models as well.

In this study, we set out to better understand the involvement of K-RAS as well as of the MAPK and PI3K signaling pathways in tumor maintenance of pancreatic cancer models *in vivo*. We developed an inducible K-RAS knock down system which allowed us to confirm requirement of pancreatic tumor maintenance on K-RAS. Having shown K-RAS dependence of our model system, we next tested involvement of the MEK or PI3K pathways in the maintenance of K-RAS dependent tumors. Response to MEK or PI3K inhibition was tested in three selected xenograft models, all of which showed tumor regression upon MEK inhibitor treatment, but not upon PI3K inhibitor treatment. Thus, all pancreatic models tested were more dependent on MAPK than on PI3K signaling. As PI3K plays important roles in regulating the tumor stroma, combined inhibition of MEK and PI3K might prove beneficial to single agent treatment despite minor effects of PI3K inhibition on tumor growth. Indeed, combining MEK and PI3K inhibitors led to superior effects compared to single agent treatment.

## Results

### K-RAS is Required for Tumor Maintenance *in vivo*

**Figure 1:**
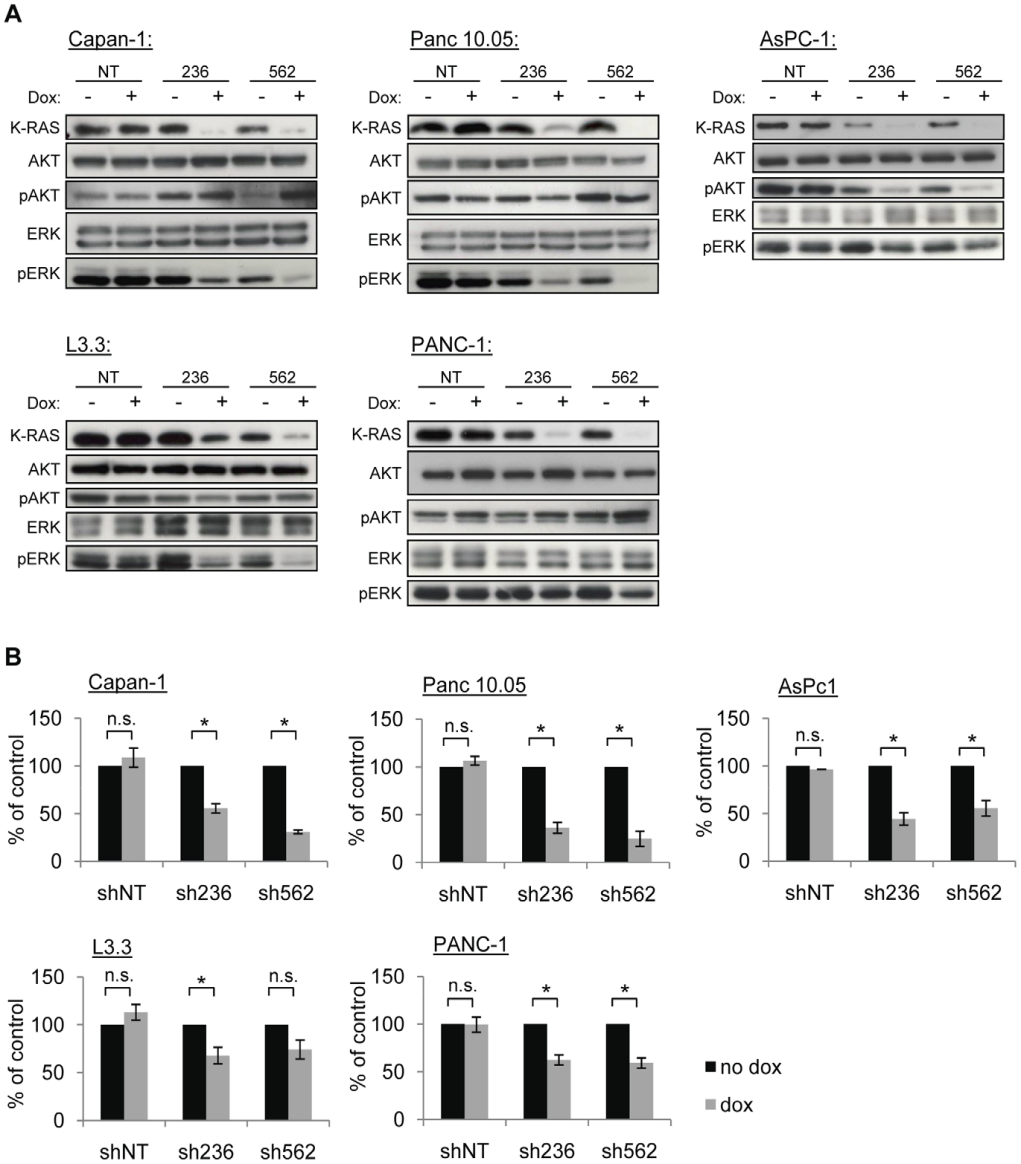
K-RAS knock down impairs proliferation in pancreatic lines *in vitro*. (A) Indicated cell lines (NT: non-targeting shRNA; 236 and 562: shRNAs targeting K-RAS) were either treated for 7 days with 200 ng/ml doxycycline (dox) or left untreated (no dox), followed by preparation of cell lysates. Corresponding cell extracts were then analyzed for K-RAS, total AKT, pAKT (Ser473), total ERK or pERK (Thr202/Tyr204) levels by Western Blot. (B) As in (A), except that cells were fixed on day 1 and day 7, followed by determination of relative cell number. Each cell line was tested in at least two independent experiments, and untreated samples were set to 100% of growth. Statistically significant differences (p<0.05) are indicated (*). Obtained p-values were as follows: Capan-1 shNT: p = 1, Capan-1 sh236: p = 0.002, Capan-1 sh562: p = 0.029; Panc 10.05 shNT: p = 0.33, Panc 10.05 sh236: p<0.001, Panc 10.05 sh562: p = 0.029; AsPc1 shNT: p = 0.33, AsPc1 sh236: p = 0.002, AsPc1 sh562: p = 0.029; L3.3 shNT: p = 0.187, L3.3 sh236: p<0.001, L3.3 sh562: p = 0.333; PANC-1 shNT: p = 1, PANC-1 sh236: p<0.001, PANC-1 sh562: p = 0.002.

Expression of mutant K-RAS is known to be required for tumor maintenance in a genetically engineered mouse model of pancreatic cancer [6]. To expand on this study, and to confirm the relevance of the findings in human cancer models, we established doxycycline-inducible K*-*RAS shRNA expression in five K-RAS mutant human pancreatic cell lines (Capan-1, Panc 10.05, AsPC-1, L3.3 and PANC-1) (Table S1). Doxycycline treatment led to effective K-RAS knock down upon K-RAS specific sh236 and sh562 expression in all lines tested *in vitro*. In contrast, no knock down was observed in the non-targeting shRNA (shNT) control pools (Figure 1A). With the exception of the L3.3 line, for which leaky expression of sh562 resulted in increased doubling times, all five K-RAS mutant pancreatic models showed impaired growth upon expression of either sh236 or sh562 when tested in proliferation assays (Figure 1B and Figure S1). No effect on growth was observed when sh236 was expressed in the K-RAS wild type lung line NCI-H1437, despite significant reduction of K-RAS protein levels, demonstrating the specificity of K-RAS knock down (Figure S2). Overall, these data confirm previously published findings showing dependence of *in vitro* proliferation on expression of mutant K-RAS in pancreatic cell lines [27]–[29]. Next, we examined effects of K-RAS knockdown on downstream signaling, and found a robust decrease of pERK levels in the Capan-1, Panc 10.05 and L3.3 lines. With the exception of the AsPC1 line, pAKT levels were found to be almost unaffected upon K-RAS knockdown (Figure 1A).

**Figure 2:**
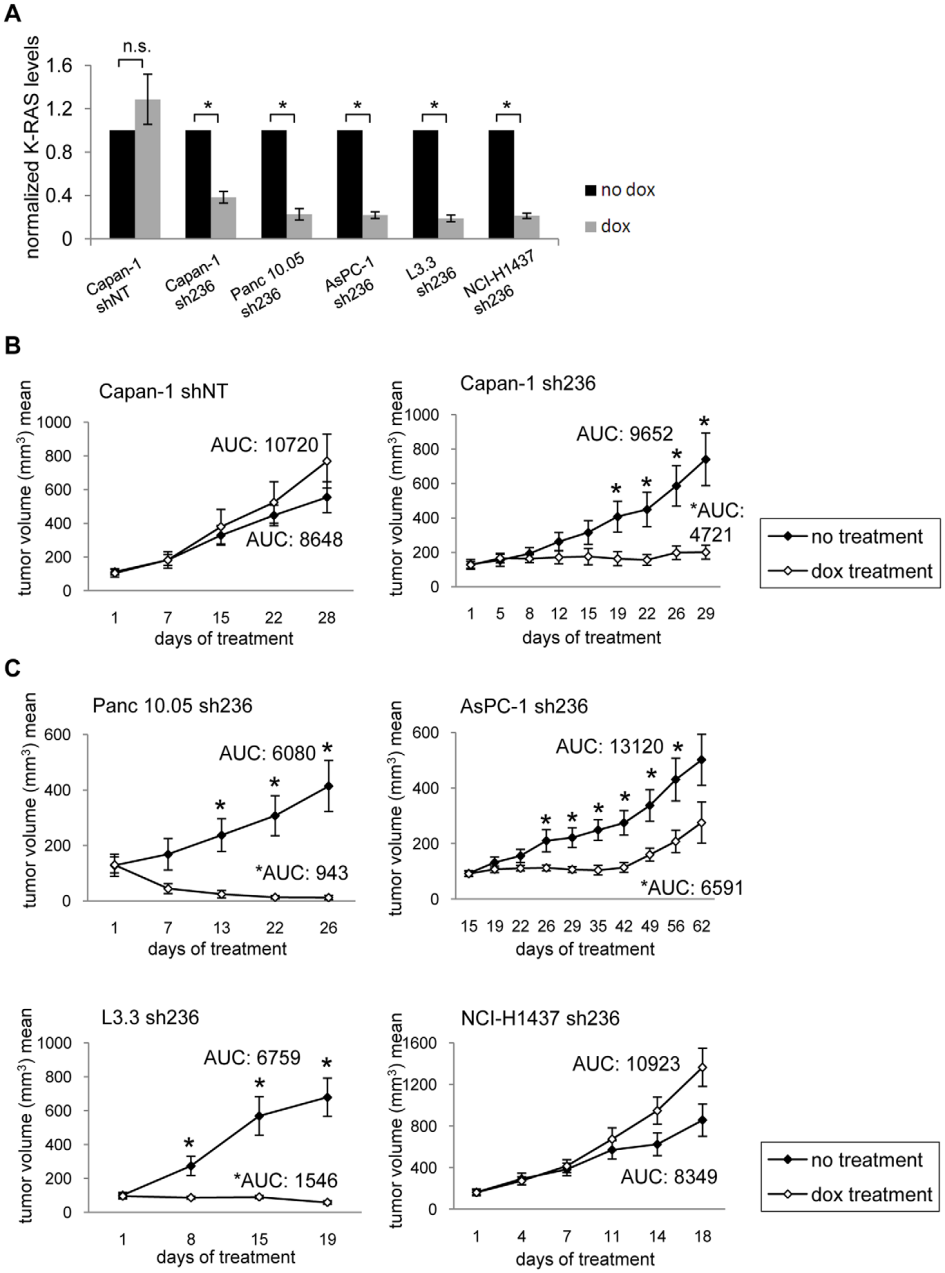
K-RAS knock down impairs tumor growth of pancreatic models *in vivo*. (A) For each xenograft model indicated, tumors were grown subcutaneously in female nude mice and groups of at least 4 mice each were formed once tumors had reached a size of 200–300 mm^3^. The first group was given normal drinking water, whereas the second was given drinking water containing 2 mg/ml doxycycline and 10% sucrose. Mice were sacrificed after one week of treatment (after 18 days in case of the K-RAS wild type model), and tumors were analyzed by qPCR for K-RAS. K-RAS levels were normalized to ribosomal protein s18. Obtained p-values were as follows: Capan-1 shNT: p = 0.35, Capan-1 sh236: p = 0.011, Panc 10.05 sh236: p = 0.009, AsPC-1 sh236: p = 0.002, L3.3 sh236: p = 0.004, NCI-H1437 sh236: p = 0.007. (B/C) As in (A), except that mice were randomized to groups of at least 6 mice each, with the exception of the Panc 10.05 model, where the group size was n = 4. Treatment was started once tumors had reached a size of 100 mm^3^, tumor size was followed over time, and mice were sacrificed once tumors of the control group reached a size of 1000 mm^3^ at most. Statistically significant differences of tumor volumes between groups (*) as well as the area under the curve (AUC/mm^3^ x treatment days) are indicated. Obtained p-values for AUC at the end of the study were as follows: Capan-1 shNT: p = 0.57, Capan-1 sh236: p = 0.04, Panc 10.05 sh236: p = 0.01, AsPC-1 sh236: p = 0.01, L3.3 sh236: p = 0.0003, NCI-H1437: p = 0.22. The PANC-1 cells could not be grown *in vivo*, and for this reason this model was only examined *in vitro*.

We next tested K-RAS dependence *in vivo* by performing nude mouse xenograft studies with four out of the five human K-RAS mutant lines (Capan-1, Panc10.05, AsPC-1 and L3.3) described above, as well as for the wild type K-RAS control line NCI-H1437. The functionality of the K-RAS knock down system in these models was first assessed by treating tumor-bearing mice with doxycycline for 7 days. This resulted in a 60 to 80% reduction of K-RAS transcript levels upon expression of shRNA236, in contrast to the non-targeting shRNA control (Figure 2A). Hence, this system is suitable for studying the role of K-RAS expression in already established tumors. Long-term doxycycline treatment of sh236 Capan1 tumor bearing mice resulted in significant antitumor activity leading to tumor stasis. No antitumor activity was observed in shNT Capan1 tumor bearing mice, showing that inhibition of tumor growth is not caused by unspecific effects of doxycycline treatment (Figure 2B). Similar studies were performed with the Panc 10.05, AsPC-1 and L3.3 models, for which doxycycline treatment resulted in tumor growth inhibition in all three cases (Figure 2C). The effects were always statistically significant when the tumor volumes across the entire study were considered by calculating the area under the curve (AUC) (Figure 2B/C and Table S2). Ablation of K-RAS expression in the NCI-H1437 tumors, however, did not result in impaired tumor growth, and thus shows K-RAS independence of this model for tumor maintenance *in vivo* (Figure 2A and 2C). In conclusion, we showed that mutant K-RAS is required for tumor maintenance of the pancreatic lineage in an *in vivo* xenograft system using human cancer cell lines.

### K-RAS Signals via MAPK *in vivo*

**Figure 3:**
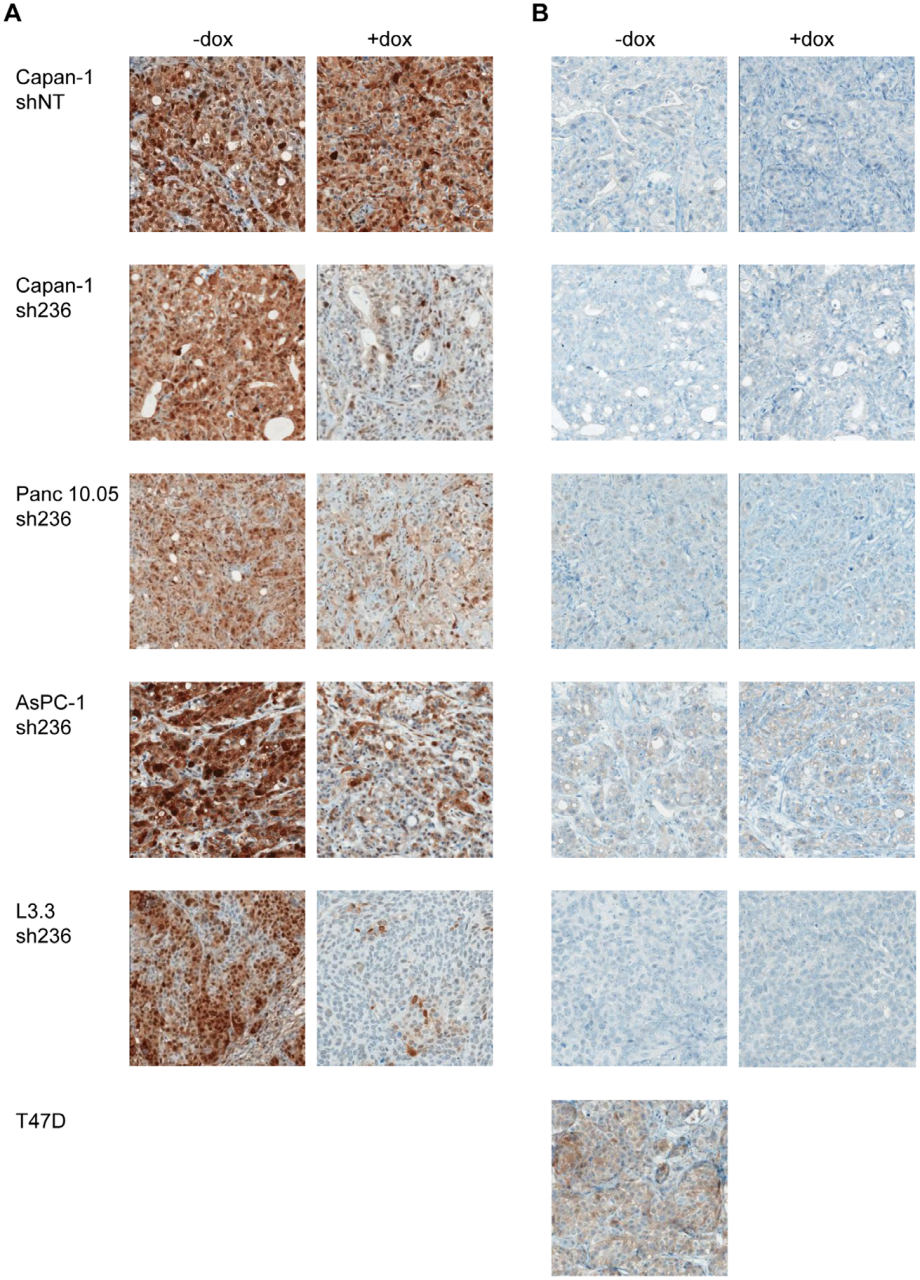
K-RAS knock down results in decreased pERK levels *in vivo*. For each xenograft model indicated, tumors were grown subcutaneously in female nude mice and groups of at least 4 mice each were formed once tumors had reached a size of 200–300 mm^3^. The first group was given normal drinking water (-dox), whereas the second was given drinking water containing 2 mg/ml doxycycline and 10% sucrose (+dox). After one week of treatment, mice were sacrificed and the tumors were removed and processed for immunohistochemistry for either pERK (Thr202/Tyr204) (A), or pAKT (Ser473) (B). The T47D model was used as an AKT dependent control model with physiological pAKT levels.

Tumor-stroma interactions have been shown to play a critical role in pancreatic cancer [6], [20]–[21], [30]. Hence, signaling events specific to the tumor environment can not be captured in *in vitro* systems. Our K-RAS dependent model system allows *in vivo* investigation of downstream signaling pathways employed by mutant K-RAS. pERK and pAKT levels were examined as readout for MAPK and PI3K pathway activity respectively by immunohistochemistry, as this has the advantage to allow discrimination of tumor tissue from tumor stroma. Basal pERK levels were readily detectable and were substantially decreased after 7 days of K-RAS shRNA expression in all four models (Capan-1, Panc 10.05, AsPC-1 and L3.3), whereas no changes were observed when the non-targeting shRNA was expressed. Hence, ERK is activated downstream of K-RAS in these tumor models (Figure 3A).

In sharp contrast, basal pAKT levels were found to be very low - almost undetectable - in all tumors tested. As positive control, pAKT expression was also determined and shown to be detectable in sections of the PI3K mutant, AKT-dependent breast cancer T47D tumors. (Figure 3B) [31]. It has to be noted that the effect of K-RAS knockdown on downstream phosphoprotein levels does not completely correlate between *in vivo* and *in vitro* settings, with pERK levels being affected strongly across all *in vivo* models (Figure 1A).

**Figure 4:**
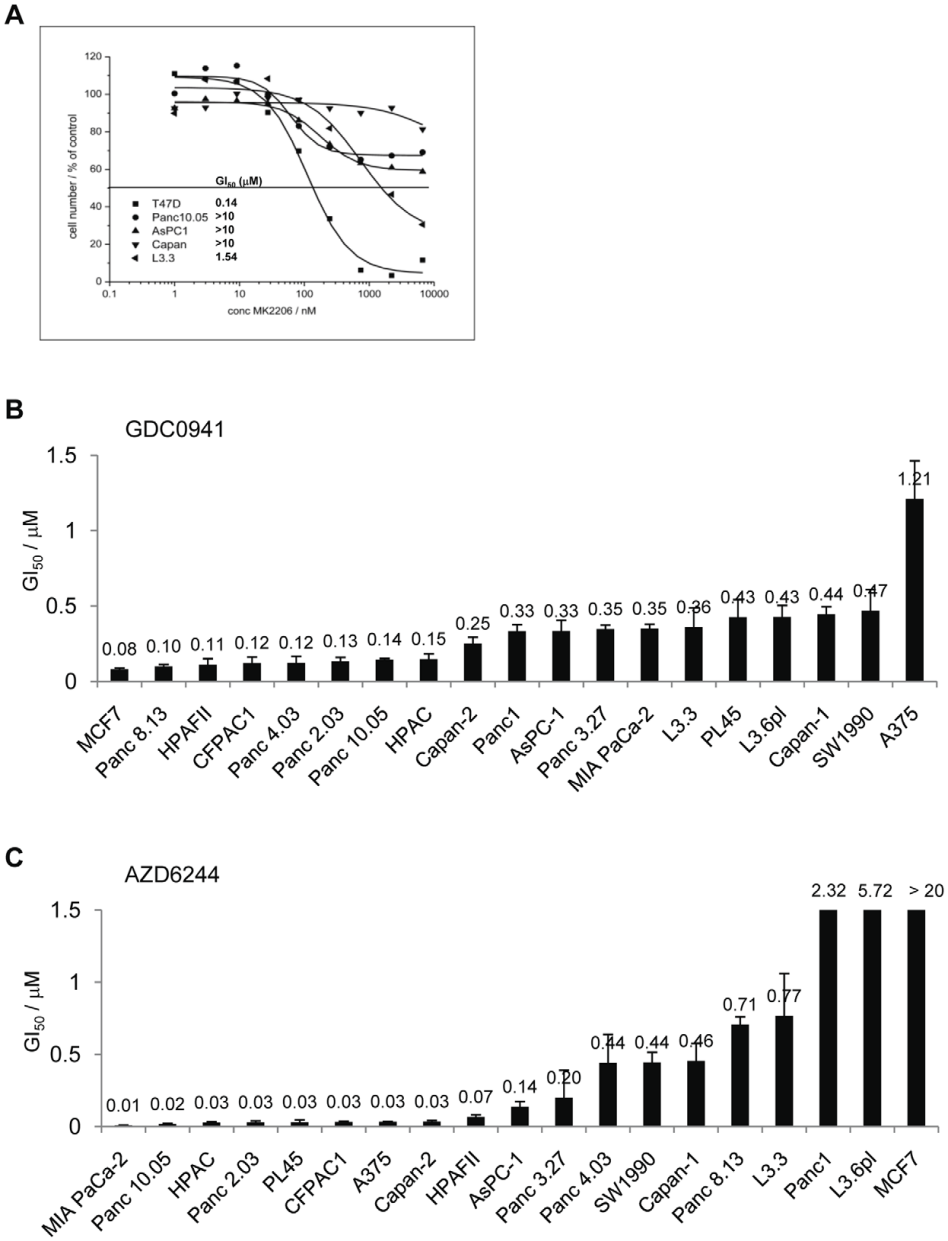
K-RAS mutant pancreatic lines are independent of AKT *in vitro*. (A). Indicated cell lines were treated for 72 h with the AKT inhibitor MK2206, and effects on proliferation were determined by calculation of respective GI_50_ values. (B/C). As in (A), except that indicated cell lines were treated for 62 h with either the PI3K inhibitor GDC0941 (B) or with the MEK inhibitor AZD6244 (C), and effects on proliferation were determined by calculation of respective GI_50_ values. MCF7 cells were used as control for cells sensitive to GDC0941 and insensitive to AZD6244 and A375 cells were used as control for cells sensitive to AZD6244 and insensitive to GDC0941.

To exclude the possibility that even such low levels of pAKT could be sufficient to promote physiologically relevant signaling, sensitivity to the AKT inhibitor MK2206 was tested *in vitro* [32]. As expected, the control line T47D was sensitive to MK2206, reflected by nanomolar GI_50_ values (GI_50_ = 140 nM). In contrast, no significant effect of AKT inhibition was seen on the pancreatic cell lines *in vitro*, with GI_50_ values of above 10 µM for the Capan1, Panc10.05 and AsPc1 lines, and a GI_50_ of 1.54 µM for the L3.3 line (Figure 4A).

In addition, we tested response of a larger panel of K-RAS mutant pancreatic cell lines to the PI3K inhibitor GDC0941 and to the MEK inhibitor AZD6244 [33]-[35]. GI_50_ values of a few pancreatic lines were close to the GI_50_ = 80 nM observed for the sensitive line MCF7, however, none of the pancreatic lines was as sensitive as the line MCF7 (Figure 4B). In contrast, 50% of lines tested for sensitivity to the MEK inhibitor AZD6244 showed GI_50_ values comparable to the values obtained for the sensitive line A-375 (GI_50_ = 34 nM) (Figure 4C). Thus, a substantial number of K-RAS mutant pancreatic lines were sensitive to MEK inhibition *in vitro*.

### Pancreatic Models Show Higher Sensitivity to MEK than to PI3K Inhibition *in vivo*

**Figure 5:**
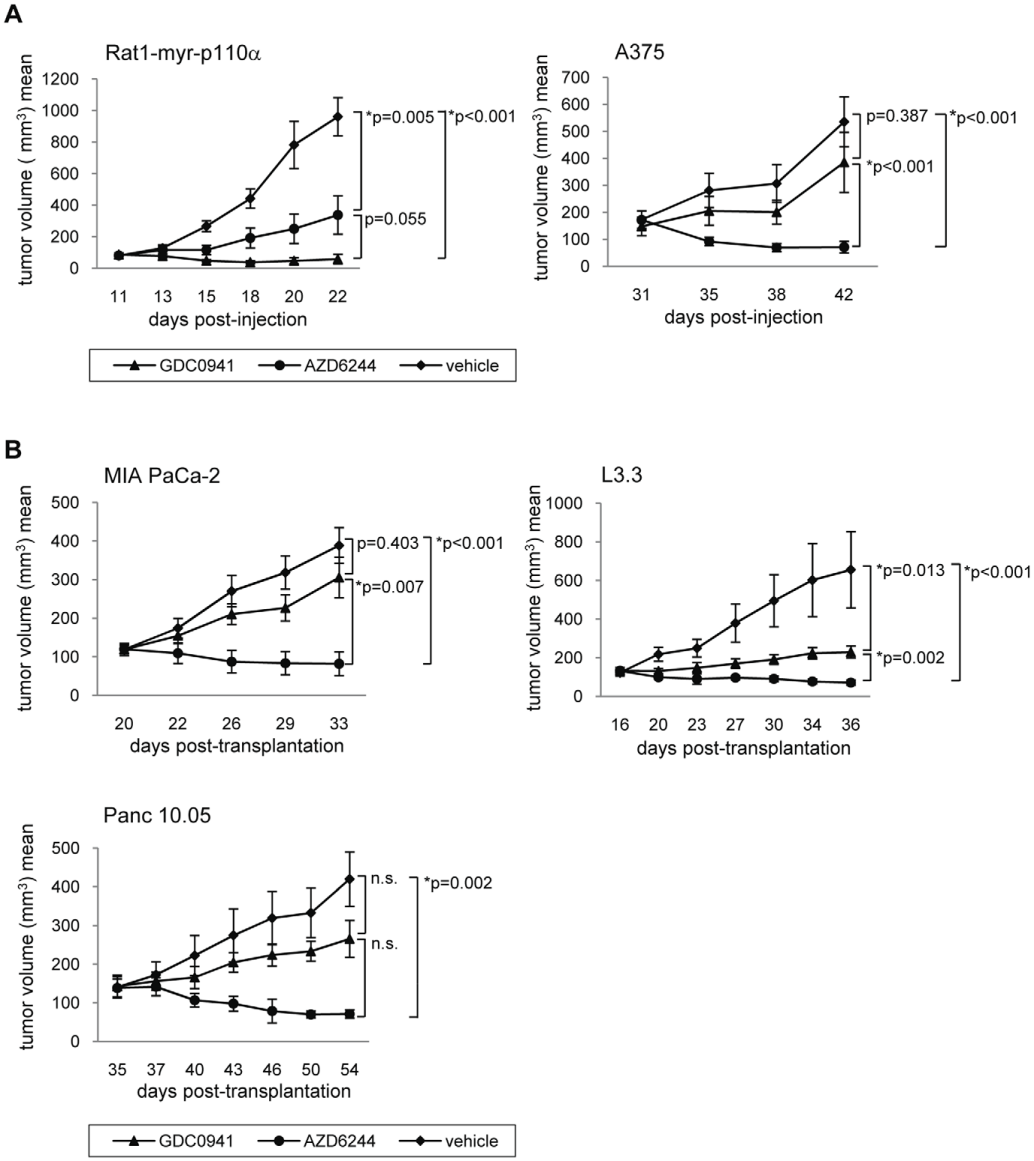
K-RAS mutant pancreatic models show stronger response to MEK than to PI3K inhibition *in vivo*. (A/B). Indicated tumor-bearing mice were treated either with GDC0941 100 mg/kg p.o. once a day, or with AZD6244 50 mg/kg p.o. twice a day, or with vehicle control, with at least 5 mice per group. Tumor volumes were measured twice a week for the indicated period of time, and antitumor activity was plotted and quantified.

**Figure 6:**
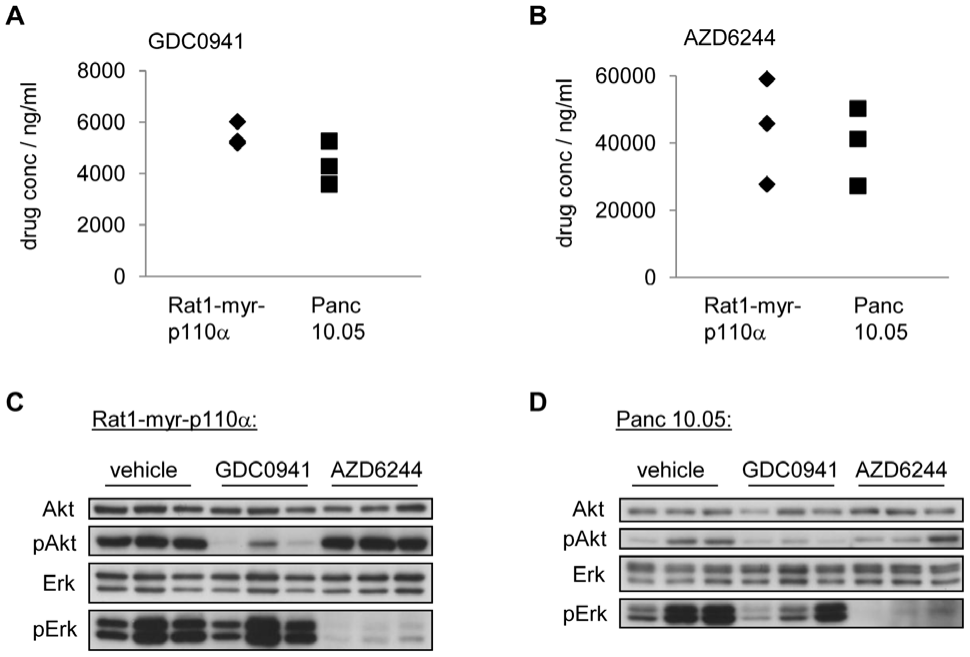
GDC0941 and AZD6244 *in vivo* treatment inhibits pAKT and pERK respectively. Indicated tumor-bearing mice were treated with a single dose of either GDC0941 100 mg/kg p.o., or AZD6244 50 mg/kg p.o., or with vehicle control. Animals were sacrificed 1 h after treatment, plasma samples were collected, analyzed and quantified by mass spectrometry for GDC0941 (A) or AZD6244 (B). Tumors were excised and analyzed by Western Blot for total AKT, pAKT (Ser473), total ERK or pERK (Thr202/Tyr204) for the model Rat1-myr-p110α (C) or the model Panc 10.05 (D).

Having shown K-RAS dependence of the xenograft models, the question as to the role of the downstream pathways MAPK and PI3K in tumor maintenance arises. Selected nude mouse xenograft models were tested for antitumor response to the PI3K inhibitor GDC0941 or the MEK inhibitor AZD6244. Rat1-myr-p110α tumors were used as control for PI3K dependence, whereas A-375 tumors were our control for MEK dependence. As expected, Rat1-myr-p110α tumors showed slight tumor regression upon treatment at the reported efficacious dose level of the PI3K inhibitor GDC0941 (T/C = −3%), but tumors did not regress upon treatment with the MEK inhibitor AZD6244 (T/C = 29%) [36]. In contrast, A-375 tumors, harboring an activating B-RAF mutation, responded strongly to AZD6244 (T/C = −28%), but did not show significant sensitivity to GDC0941 (T/C = 66%) (Figure 5A). We then tested the response of three pancreatic models (MIA PaCa-2, L3.3 and Panc 10.05). The MIA PaCa-2 model was included because the cell line showed the highest sensitivity to MEK inhibition amongst the pancreatic lines tested *in vitro* (Figure 4C). Interestingly, all three models displayed statistically significant tumor regression upon treatment with AZD6244 (T/C(MIA PaCa-2) = −14%, T/C(L3.3) = −12%, T/C(Panc 10.05) = −24%), while modest growth inhibition but no tumor regression was observed in response to GDC0941 treatment (T/C(MIA PaCa-2) = 69%, T/C(L3.3) = 18%, T/C(Panc 10.05) = 44%) (Figure 5B). It has to be noted that *in vivo* efficacy correlated poorly with *in vitro* sensitivity, and so *in vitro* data might not be useful for predicting the *in vivo* response of pancreatic cancer models (Figure 4B and C). Drug plasma levels were comparable between the Rat1-myr-p110α and the Panc 10.05 models after a single treatment, indicating sufficient absorption of either compound (Figure 6A and B). Moreover, respective targets were found to be inhibited in both models, with very low pERK and pAKT levels following drug exposure. As expected, basal pAKT levels were low in the pancreatic model Panc 10.05, and so inhibition of pAKT seemed less pronounced compared to the high pAKT-expressing model Rat1-myr-p110α (Figure 6C and D). In conclusion, all three pancreatic models tested *in vivo* showed higher dependence on MAPK than on PI3K signaling, indicating that it is the MAPK pathway playing the major role in tumor maintenance.

### Combining MEK and PI3K Inhibition *in vivo* is Superior to Single Agent Treatment

**Figure 7:**
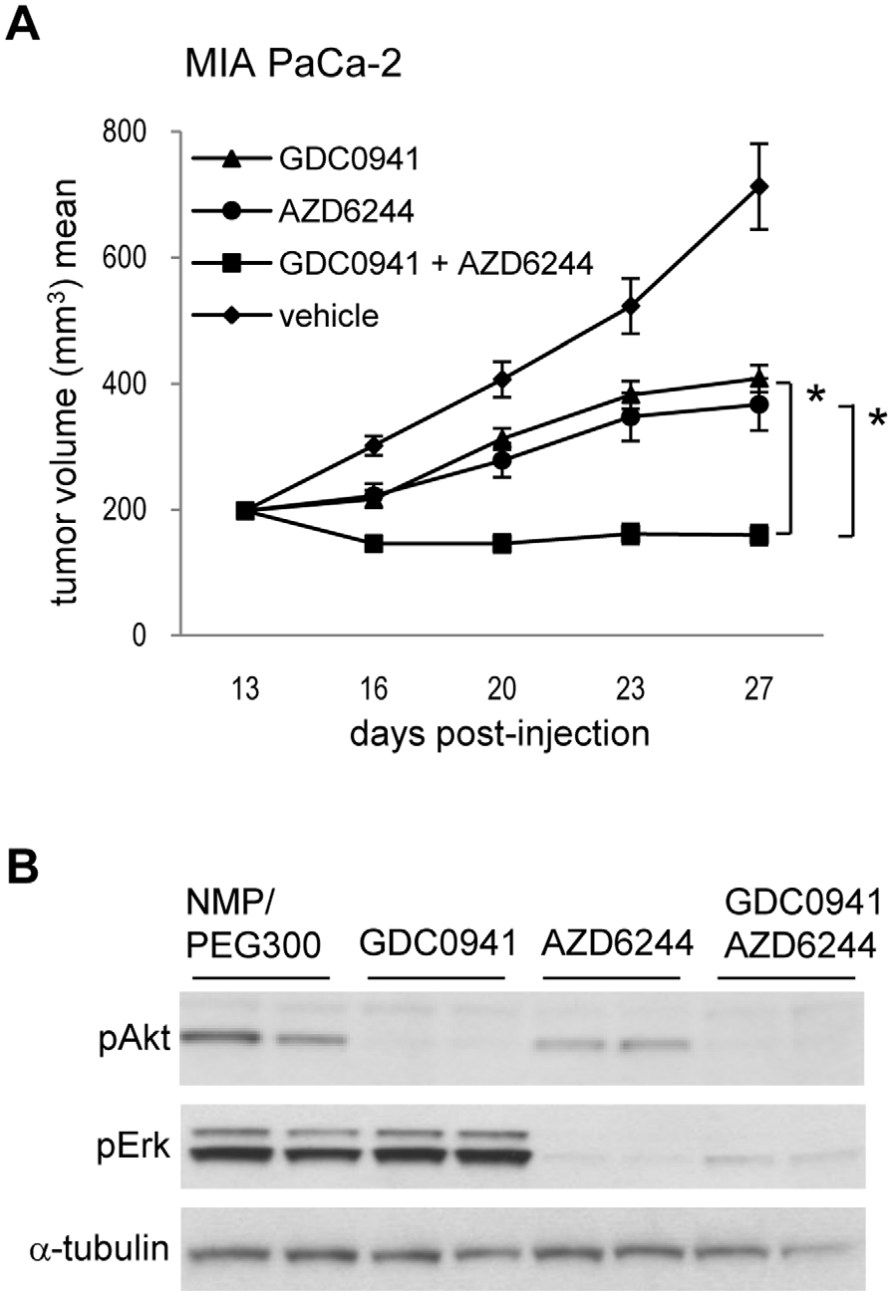
Combining MEK and PI3K inhibition *in vivo* is superior to single agent treatment. (A). Indicated tumor-bearing mice were treated either with GDC0941 100 mg/kg p.o. once a day, or with AZD6244 5 mg/kg p.o. once a day, or with the combination of both, or with vehicle control, with 8 mice per group. Tumor volumes were measured twice a week, for the indicated period of time, and antitumor activity was plotted and quantified. (B). Indicated tumor-bearing mice were treated with a single dose of either GDC0941 100 mg/kg p.o. or of AZD6244 5 mg/kg p.o., with the combination of both or with vehicle control. Animals were sacrificed 3 h after treatment, tumors were excised and analyzed by Western Blot for total AKT, pAKT (Ser473), total ERK or pERK (Thr202/Tyr204).

A number of studies have reported synergy for combined use of MEK and PI3K inhibitors in K-RAS mutant breast, lung and colorectal tumor models [14]–[15], [24]–[26]. PI3K inhibition had limited effect on tumor growth in the pancreatic models tested, however, PI3K has well described functions in the tumor stroma of pancreatic cancers, and therefore combined application of a PI3K and a MEK inhibitor might prove beneficial by targeting both tumor cells as well as stromal cells [20]. As expected, treatment of nude mice bearing MIA-PaCa-2 tumors with the PI3K inhibitor GDC0941 alone resulted in limited tumor growth inhibition (T/C = 41%). Treatment with the MEK inhibitor AZD6244 alone was done at a lower dose of 5 mg/kg and led to a similar tumor growth inhibition with a T/C of 33%. Notably, combining GDC0941 and AZD6244 showed synergistic tumor regression with a T/C of −20% (Figure 7A). pAKT and pERK were found inhibited upon exposure to a single dose of respective compound, as well as upon combination treatment (Figure 7C). To test the effect of the MEK/PI3K combination on a second K-RAS mutant pancreatic xenograft model, nude mice bearing Panc 10.05 tumors were treated with AZD6244, GDC0941 or the combination of both. Treatment with either inhibitor alone resulted in tumor growth inhibition with a T/C of 47% upon AZD6244 application and a T/C of 12% upon GDC0944 application. As observed for the MIA PaCa-2 model, combination of AZD6244 and GDC0941 led to tumor regression with a T/C of −33% (Figure S3). Thus, combining MEK and PI3K inhibitors is superior to single agent treatment in two *in vivo* models of the pancreatic lineage.

## Discussion

Patients with advanced pancreatic ductal adenocarcinoma (PDAC) are commonly treated with the chemotherapeutic gemcitabine. As 5 year survival rates are very low (<5%), new therapies are clearly needed [37]. Genetic mouse models have helped to understand the crucial role of activating K-RAS mutations in the onset and maintenance of pancreatic cancer [3]–[6]. To investigate K-RAS dependent tumor maintenance of human cell lines, we generated an inducible K-RAS shRNA knock down system which allowed us to ablate K-RAS expression in established tumors. In all four pancreatic xenograft models studied, we observed impaired tumor growth upon K-RAS knock down. Thus, as in the genetic mouse model, K-RAS is required for tumor maintenance of human xenografts of the pancreatic lineage *in vivo*.

No specific inhibitors targeting K-RAS have been developed to date, and so the identification of the key effectors mediating tumor maintenance might lead to alternative therapeutic opportunities. Such downstream targeting has the caveat that the oncogene itself stays active and inhibition might therefore not be complete. As all attempts to target K-RAS have failed so far, targeting downstram signaling pathway seems a promising alternative at present [7]. Notably, all three pancreatic xenograft models tested *in vivo* showed regression upon MEK, but not upon PI3K inhibition. This indicates higher dependence of established pancreatic tumors on MAPK than on PI3K signaling. Similar results have been described for K-RAS induced lung tumors, with MEK but not PI3K inhibition leading to tumor regression [14]–[15]. Therefore, MAPK signaling might - in addition to its prominent role in the lung - also play a major role in the maintenance of pancreatic tumors. Future studies will be needed to understand if this might be a more general phenomenon across K-RAS mutant tumors.

At present, the mechanism explaining the stronger response to MEK than to PI3K inhibition in the pancreatic xenografts examined is not known. We showed K-RAS to signal via MAPK, and so it is tempting to speculate that sensitivity to MEK inhibitors is linked to pathway activity in these models. A few *in vivo* models of K-RAS mutant pancreatic cancers have been described to be sensitive to MEK inhibition, whereas K-RAS mutations have been shown to be predictive of resistance to treatment with PI3K inhibitors in several tumor types [17]–[19]. The mechanism of insensitivity to PI3K inhibition was not further elucidated in these publications, and future studies will be required to gain such insight. None of these studies have directly compared response to MEK versus PI3K inhibition.

Inhibition of PI3K actually resulted in tumor growth inhibition in the model L3.3, though to a less dramatic extent than upon MEK inhibition. As was the case for all pancreatic models tested, the L3.3 model showed low pAKT levels and independence of AKT signaling. PI3K signaling appears to depend upon PDK1 rather than AKT in several breast cancer cell lines harboring the H1047R mutation in PIK3CA, and thus it remains to be seen if a similar mechanism exists in the L3.3 model [38]. Moreover, the L3.3 line is wild type for p53, whereas all other lines tested *in vivo* harbor mutations in the gene. It would be interesting to investigate if there is a link between p53 status and response to PI3K inhibition.

A number of PI3K and MEK inhibitors are currently being developed and tested in clinical studies [39]–[40]. PI3K inhibitors have been tested in phase I studies in patients with solid tumors with promising outcomes [41]. However, no phase II data is available yet, and thus future studies need to be awaited to conclude upon the effectiveness of such inhibitors. AZD6244 has been tested in a phase II study in patients with advanced pancreatic cancer, with the outcome of no statistically significant difference in overall survival between AZD6244 and standard of care [42]. This result is in disagreement to our data, showing regression of K-RAS mutant pancreatic tumors in xenograft models. It must be noted that the clinical trial was performed as second-line treatment on a patient population with advanced pancreatic cancer who have failed first-line gemcitabine therapy. It is possible that MEK inhibition as first-line treatment for pancreatic cancer might prove to be more effective. *In vitro*, MEK inhibition was not effective in a gemcitabine resistant pancreatic line established by exposure to the chemotherapeutic [43]. Furthermore, hypoxia has been used to induce gemcitabine resistance in pancreatic cell lines, and such cells proved to be unresponsive to MEK inhibition. Interestingly, the same cell line was sensitive to gemcitabine as well as to MEK inhibition under normoxic conditions [44]. These data indicate that MEK inhibitors might indeed not be effective on gemcitabine resistant cells. Such gemcitabine resistance could be reversed in a genetic mouse model of pancreatic cancer by inhibition of the hedgehog pathway, which mediated remodeling of the tumor stroma and thus facilitated uptake of gemcitabine into the tumor [45]. Hence one could speculate that a PI3K inhibitor as well as other modalities might have similar effects on the stromal compartment, leading to increased drug penetration of the tumor and in this way to increased gemcitabine sensitivity [20], [46]–[49].

Moreover, target inhibition in the tumors was not determined in this phase II study, and it is possible that pERK levels were not sufficiently decreased to show efficacy. Tumors generally show vascular abnormalities, with dilated, irregular vessels which are poorly functional [50]. Pancreatic tumors are known to be hypoperfused and therefore show limited uptake of drugs [51]. This phenomenon is less prevalent in the case of transplanted tumors like the xenograft models used in our study [45]. Thus, it is possible that MEK inhibition had limited success in above mentioned trial because the drug could not sufficiently enter the tumors.

Targeting the tumor stroma in a genetic mouse model of pancreatic cancer led to changes in the tumor vasculature which allowed increased uptake of the drug into the tumor, resulting in improved efficacy [45]. A number of publications have shown that GDC0941 as well as other PI3K inhibitors lead to remodeling of the tumor vasculature, resulting in increased drug uptake [46]–[49]. Combined application of PI3K inhibitors might therefore be generally beneficial in enhancing the delivery of drugs into the tumor. When we treated nude mice bearing MiaPaCa-2 tumors - which showed limited sensitivity to PI3K inhibition *in vivo* - with a combination of a MEK and a PI3K inhibitor, we observed an effect superior to MEK inhibition alone. A second xenograft model, mice bearing Panc 10.05 tumors, responded similarly and showed benefit of combined administration of a MEK and a PI3K inhibitor. This indicates that K-RAS mutant pancreatic xenografts might generally show superior response upon MEK/PI3K inhibitor combination treatment. The mechanism of this synergy has not been investigated, however, it is possible that the combination is beneficial by targeting both tumor cells and tumor stroma. Future studies are clearly needed to support this hypothesis, and to investigate if PI3K inhibition aids the uptake of the MEK inhibitor into the tumor.

Combination treatment of K-RAS mutant breast, lung and colorectal tumors with a MEK and a PI3K inhibitor has been shown to be superior to single agent treatment. Frequently, the combination led to enhanced induction of apoptosis [14]–[15], [24]–[26]. Moreover, resistance to MEK inhibition was found to be mediated by activation of PI3K signaling in several lineages, and inhibition of both pathways showed synergistic effects [22]–[23]. It remains to be seen whether a similar resistance mechanism takes place in pancreatic tumors; the existence of which would provide better understanding of the synergy seen with the PI3K and MEK inhibitor combination.

Our data on combining MEK and PI3K inhibition in pancreatic xenograft models supports use of this combination for future clinical trials. Indeed, such combination trials are currently being prepared, and the results of these are eagerly awaited with the hope that such treatment will result in improved responses in the clinic.

## Materials and Methods

### Ethics Statement

All animal experiments were fully approved by the Kantonales Veterinäramt Basel-Stadt under license #1769 and were conducted in accordance with the Eidgenössisches Tierschutzgesetz and the Eidgenössische Tierschutzverordnung.

### Chemical Compounds

GDC0941, AZD6244 and MK2206 were obtained from Selleck Chemicals, Boston, USA.

### Cell Lines and Cell Culture

Cell lines were purchased from the American Type Cell Collection (Manassas, USA). All lines were cultured at 37°C, 5% CO_2_ and 80% relative humidity in DMEM high glucose (Gibco, Carlsbad, USA) complemented with 10% fetal bovine serum (20% in case of the cell line Capan1), 2 mM glutamine and 1% penicillin-streptomycin.

### Cell Lysate Preparation and Immunoblotting

Cells were washed with cold PBS and lysed in 1% NP40 lysis buffer. Lysates were centrifuged for 10 min at 13000 rpm to remove cellular debris, and the protein concentration was determined using the Bradford test. Tumor lysates were prepared by homogenizing the tumors, resuspending the powder in 1% NP40 lysis buffer followed by a centrifugation step for 10 min at 13000 rpm and determination of the protein concentration. Western blotting was done on PVDF membranes using PBS/Tween (0.1%) milk and the following antibodies: AKT (Epitomics, Burlingame, USA; #1085-1, 1∶1000), pAKT (Ser473; CST, Canvers, USA; #9271, 1∶1000), ERK (CST #9102, 1∶1000), pERK (Thr202/Tyr204; CST #9101, 1∶1000), and K-RAS (Santa Cruz Biotechnology, Santa Cruz, USA; #sc−30, 1∶200). HRP labeled secondary antibodies were detected using ECL (Amersham Biosciences, Little Chalfont, UK) and autoradiography.

### Lentivirus Production and Infection

HEK 293FT cells were transfected with DNA-Lipofectamine complexes (Invitrogen, Carlsbad, USA) containing pVPRΔ8.71, pVSVG and LKO-Tet-ON vector [52]. The next day, 1 mM sodium pyruvate and 10 mM sodium butyrate were added to the medium for 8 h. Virus was harvested 24 h later, filtered and titrated in MIA PaCa-2 cells.

Cells were spinfected at 2000 rpm for 2 h in medium containing Tet-free FCS and 8 µg/ml polybrene at an MOI = 1. Medium was changed 8–16 h post-infection, and puromycin selection was started and maintained after 30 h of recovery at 1 µg/ml. Target sequences of shRNAs: K-RAS sh236: 5′ GATACAGCTAATTCAGAATC 3′; K-RAS sh562: 5′ AGGCTCAGGACTTAGCAAGA 3′; shNT: 5′ GGATAATGGTGATTGAGATGG 3′.

### Proliferation Assay

Cells were plated in 96 well plates with 6 replicates per condition. The next day, doxycycline was added at 200 ng/ml and changed every 3 days. Cells were fixed in glutaraldehyde at indicated days, stained in methylene blue, washed, the dye was eluted in 3% HCl, and the plates were read at OD = 650 nm. Statistics were calculated by performing a t-test; p-values <0.05 were considered statistically significant. In cases where the equal variance or the normality test failed, a Whitney- Mann test was performed.

For determination of GI_50_ values, cell lines were plated in 96 well plates. The next day, cells were treated with the AKT inhibitor MK2206 at compound concentrations ranging from 10 µM to 1 nM (from 20 µM to 1 nM for treatment with the PI3K inhibitor GDC0941 or the MEK inhibitor AZD6244). After an incubation of 72 h, cells were fixed and stained as described above. Conditions were done in duplicate, and at least 2 independent experiments were performed for each cell line.

### qPCR

RNA was isolated from frozen tumor powder and 2 µg RNA were reverse transcribed (Applied Biosystems, Foster City, USA). qPCR reactions were performed with 40 ng of transcribed RNA (qPCR core kit for SYBR Green, Eurogentec, Liege, Belgium) using following primers designed to be human-specific and to cross an exon-intron boundary: K-RAS: forward 5′ ctaaatcatttgaagatattcacc 3′; reverse 5′ctgatgtttcaataaaaggaattc 3′. qPCR of RPS18 was done using the TaqMan probe 4319413E (Applied Biosystems). Statistics were calculated by performing a t-test; p-values <0.05 were considered statistically significant. In cases where the equal variance or the normality test failed, a Whitney-Mann test was performed.

### Immunohistochemistry

Tumors were fixed after dissection in 10% neutral buffered formalin for 24 h at RT, rinsed in PBS, processed for dehydration, cleared and paraffinized. After embedding in paraffin, 3 µm sections were prepared. IHC staining was done on a Discovery XT platform (Ventana Medical Systems, Tucson, USA) using CC ultra mild epitope recovery conditions for pERK and CC ultra extended for pAKT. The detection system used was OmniMap DAB anti–Rabbit (HRP) detection kit (Ventana Medical Systems). Slides were hematoxylin counterstained and antibodies used were pAKT (Ser473; CST #4060, 1∶25) and pERK (Thr202/Tyr204; CST #4370, 1∶400).

### Xenograft Studies

For K-RAS knock down studies *in vivo*, female Harlan nude mice (Harlan Inc., Indianapolis, USA) were injected s.c. with 5 million cells in 100 µl HBSS, the Panc 10.05 cells were injected with 50% Matrigel. At a tumor volume of around 100 mm^3^ (200–300 mm^3^ for one-week treatments), mice were randomized to one of two groups and treated in the presence or absence of doxycycline. Doxycycline treatment was done by adding 2 mg/ml doxycycline in 10% sucrose in the drinking water. Tumor volumes were followed over time and are shown as mean volume +/− standard error of the mean. At the end of each study, tumors were excised and processed for IHC or qPCR. Tumor volumes were determined by using calipers for measurement of longest (considered as length) and shortest (considered as diameter) dimensions of each tumor and according to the formula V = (π * L * (D^2^))/6, with L = tumor length and D = tumor diameter. Statistics were calculated by performing a t-test. p-values <0.05 were considered statistically significant.

For inhibitor studies of pancreatic models *in vivo*, female Harlan nude mice were injected s.c. with 10 million cells in 100 µl HBSS, the MIA PaCa-2 and the Panc 10.05 cells were injected with 50% Matrigel. At a tumor volume of around 600 mm^3^, tumor pieces were transplanted s.c. Once these tumors were established to a size of around 100 mm^3^, mice were randomized to one of three groups. The Rat1-myr-p110α and the A-375 model were established by injecting 5 million cells s.c. GDC0941 and AZD6244 (free base) were formulated in NMP/PEG300 (10/90, V/V). GDC0941 was given at 100 mg/kg once a day p.o., AZD6244 at 50 mg/kg twice a day p.o. and NMP-PEG was given twice a day p.o. Tumor volumes were followed over time and are shown as mean volume +/− standard error of the mean. When required, tumors were excised at the end of the study and processed for Western blot, and blood was taken for PK studies. Antitumor activity is expressed as T/C% (mean increase of tumor volumes of treated animals divided by the mean increase of tumor volumes of control animals multiplied by 100).

For combination studies, female Harlan nude mice were injected s.c. with 5 million MIA PaCa-2 cells in 100 µl HBSS containing 50% Matrigel. Once tumors were established to a size of around 150 mm^3^, mice were randomized to one of four groups. GDC0941 and AZD6244 (free base) were formulated in NMP/PEG300 (10/90, V/V). GDC0941 was given at 100 mg/kg once a day p.o., AZD6244 at 5 mg/kg once a day p.o. and NMP-PEG was given once a day p.o. The combination was given at 100 mg/kg GDC0941 once a day p.o., and 5 mg/kg AZD6244 once a day p.o. Tumor volumes were followed over time and are shown as mean volume +/− standard error of the mean. Synergy was determined using the Clarke method [53].

Statistical analysis was done by a one way ANOVA Tukey test; p-values <0.05 were considered statistically significant. In case the normality or the equal variance test failed during this analysis, log tranformed data was used for the one way ANOVA Tukey test. In case of normality or equal variance tests failing for both untransformed and log transformed data, an ANOVA on ranks test was performed.

### Determination of Compound Plasma Concentrations

Mouse plasma was chromatographically separated by HPLC (Agilent, Santa Clara, USA) on a RESECT™ Ultra Phenyl reverse-phase column. Compound concentrations were determined using a Quattro Micro™ mass spectrometer (Waters, Milford, USA) by comparison to a compound standard.

## Supporting Information

Figure S1 **The L3.3 sh562 cell line shows increased doubling times.** Indicated L3.3 pools were either exposed to 200 ng/ml of doxycycline (dox) or not exposed to doxycycline (no dox) for 7 days, and relative cell numbers were quantified. The doubling time was subsequently calculated using the following formula: doubling time = t*((LN(2))/(LN(OD650-t2/OD650-t1)), with t = incubation time, OD650-t2 = OD650 after 7 days of growth, OD650-t1 = OD650 at time of doxycycline addition.
(TIF)

Figure S2 **Proliferation of the K-RAS wt line NCI-H1437 is not affected upon K-RAS knock down.** (A) NCI-H1437 cell pools (NT: non-targeting shRNA; 236: shRNA targeting K-RAS) were either treated for 7 days with 200 ng/ml of doxycycline (dox) or left untreated (no dox), followed by preparation of cell lysates. Corresponding cell extracts were then analyzed for K-RAS and total AKT levels by Western Blot. (B) As in (A), except that cells were fixed on day 1 and day 7, followed by determination of proliferation. Each cell line was tested in at least two independent experiments and untreated samples were set to 100% of growth.
(TIF)

Figure S3 **Combined application of a PI3K and a MEK inhibitor is superior to single agent treatment in the model Panc 10.05.** Indicated tumor-bearing mice were treated either with GDC0941 100 mg/kg p.o. once a day, or with AZD6244 5 mg/kg p.o. once a day, or with the combination of both, or with vehicle control, with 6 mice per group. Tumor volumes were measured twice a week, for the indicated period of time, and antitumor activity was plotted and quantified.
(TIF)

Table S1 **Mutational status of pancreatic cell lines used.** The mutational status of K-RAS, TP53, CDKN2A and SMAD4 of the panel of pancreatic cell lines was collected from the Cosmic database or from Oncomap. In cases where K-RAS mutation status was not available, sequencing was performed internally ^(NVS)^. Cell lines in bold were tested *in vivo.*
(PPT)

Table S2 **Antitumor activities obtained in pancreatic models upon K-RAS knock down.** Data shown in Figure 2B and 2C was analyzed by calculating the doubling time of the tumors, T/C (treatment/control) on day 18 (minimum treatment period), T/C on the last day of each study, and Δ tumor volume between the start and end of the study, as well as the area under the curve (AUC). Statistics were calculated by performing a t-test. No t-test calculation was possible for the doubling time of the model Capan-1 K-RAS sh236, as there was no tumor growth for 2 tumors in the doxycycline treated group resulting in infinite doubling times. An outlier determined by the Grubb’s test amongst the doubling times calculated for the model AsPC-1 K-RAS sh236 was not considered in the analysis.
(PPT)
