# Supplementary figures and images for: K-RAS Mutant Pancreatic Tumors Show Higher Sensitivity to MEK than to PI3K Inhibition In Vivo
Source: PLoS One. 2012 Aug 31;7(8):e44146. doi: 10.1371/journal.pone.0044146 (PMC3432074; doi:10.1371/journal.pone.0044146)

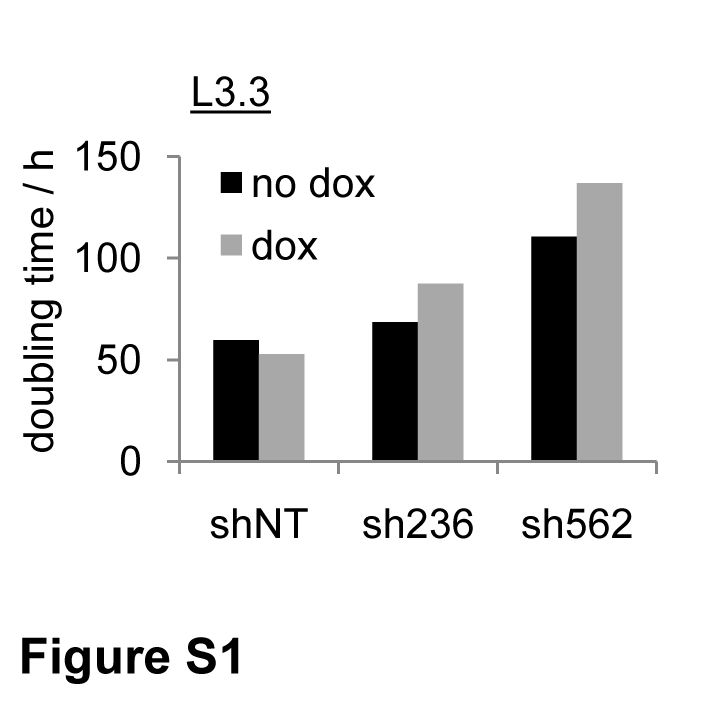

Supplement: Figure S1 — The L3.3 sh562 cell line shows increased doubling times. Indicated L3.3 pools were either exposed to 200 ng/ml of doxycycline (dox) or not exposed to doxycycline (no dox) for 7 days, and relative cell numbers were quantified. The doubling time was subsequently calculated using the following formula: doubling time = t*((LN(2))/(LN(OD650-t2/OD650-t1)), with t = incubation time, OD650-t2 = OD650 after 7 days of growth, OD650-t1 = OD650 at time of doxycycline addition. (TIF) [file pone.0044146.s001.tif]

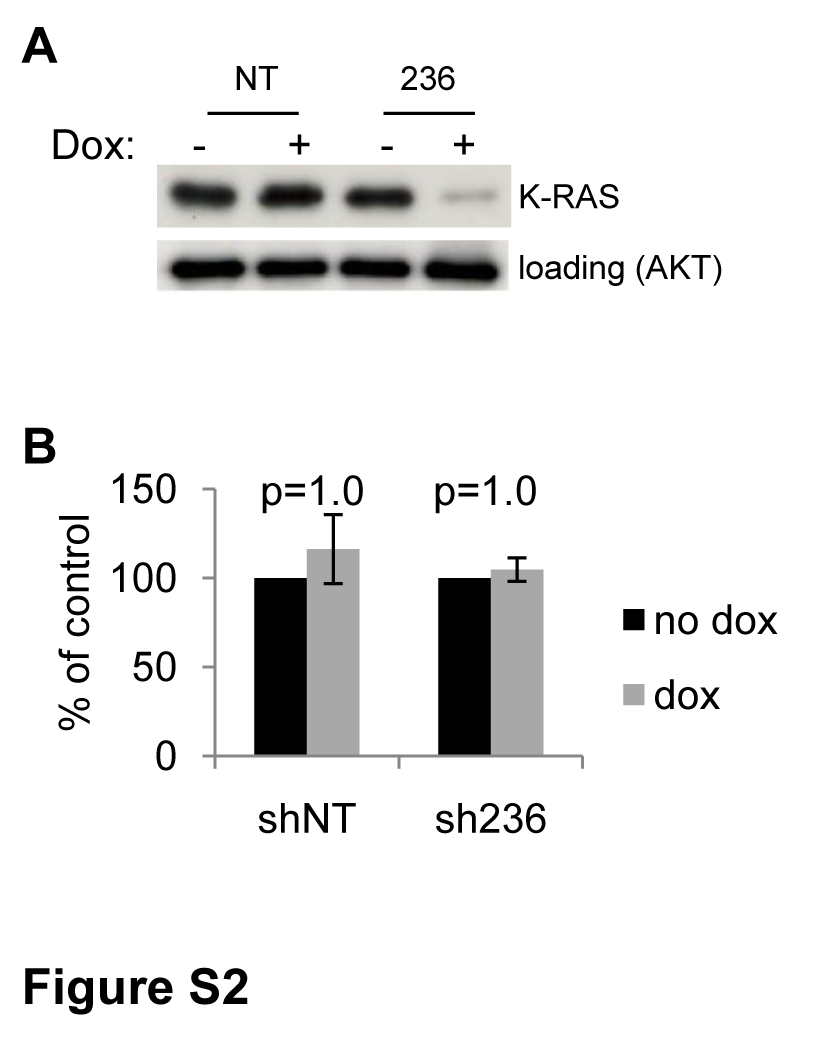

Supplement: Figure S2 — Proliferation of the K-RAS wt line NCI-H1437 is not affected upon K-RAS knock down. (A) NCI-H1437 cell pools (NT: non-targeting shRNA; 236: shRNA targeting K-RAS) were either treated for 7 days with 200 ng/ml of doxycycline (dox) or left untreated (no dox), followed by preparation of cell lysates. Corresponding cell extracts were then analyzed for K-RAS and total AKT levels by Western Blot. (B) As in (A), except that cells were fixed on day 1 and day 7, followed by determination of proliferation. Each cell line was tested in at least two independent experiments and untreated samples were set to 100% of growth. (TIF) [file pone.0044146.s002.tif]

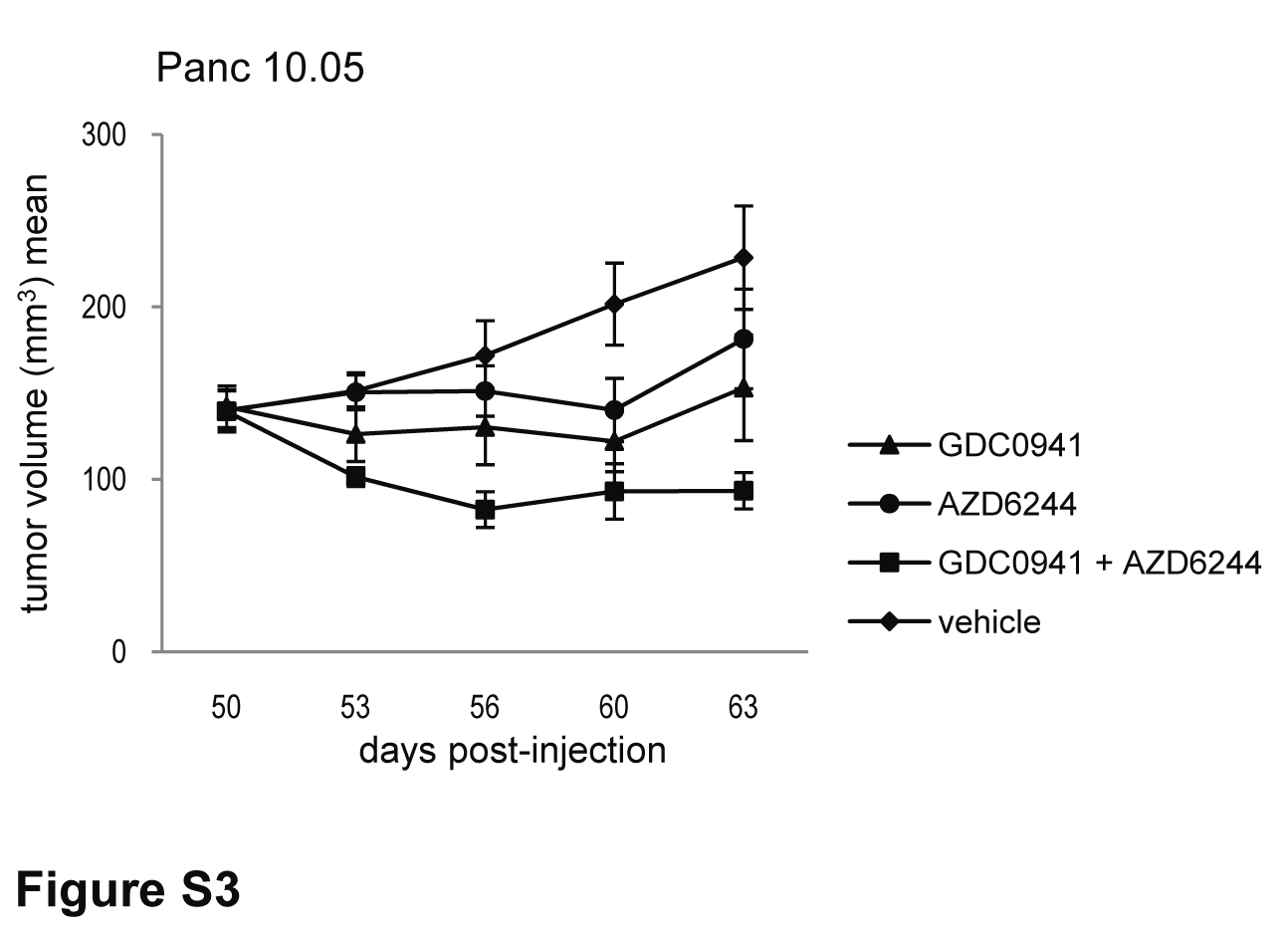

Supplement: Figure S3 — Combined application of a PI3K and a MEK inhibitor is superior to single agent treatment in the model Panc 10.05. Indicated tumor-bearing mice were treated either with GDC0941 100 mg/kg p.o. once a day, or with AZD6244 5 mg/kg p.o. once a day, or with the combination of both, or with vehicle control, with 6 mice per group. Tumor volumes were measured twice a week, for the indicated period of time, and antitumor activity was plotted and quantified. (TIF) [file pone.0044146.s003.tif]
