# Supplementary material for: K-RAS Mutant Pancreatic Tumors Show Higher Sensitivity to MEK than to PI3K Inhibition In Vivo
Source: PLoS One. 2012 Aug 31;7(8):e44146. doi: 10.1371/journal.pone.0044146 (PMC3432074; doi:10.1371/journal.pone.0044146)
Supplement: Table S1 — Mutational status of pancreatic cell lines used. The mutational status of K-RAS, TP53, CDKN2A and SMAD4 of the panel of pancreatic cell lines was collected from the Cosmic database or from Oncomap. In cases where K-RAS mutation status was not available, sequencing was performed internally (NVS). Cell lines in bold were tested in vivo. (PPT) [file pone.0044146.s004.ppt]

## Slide 1
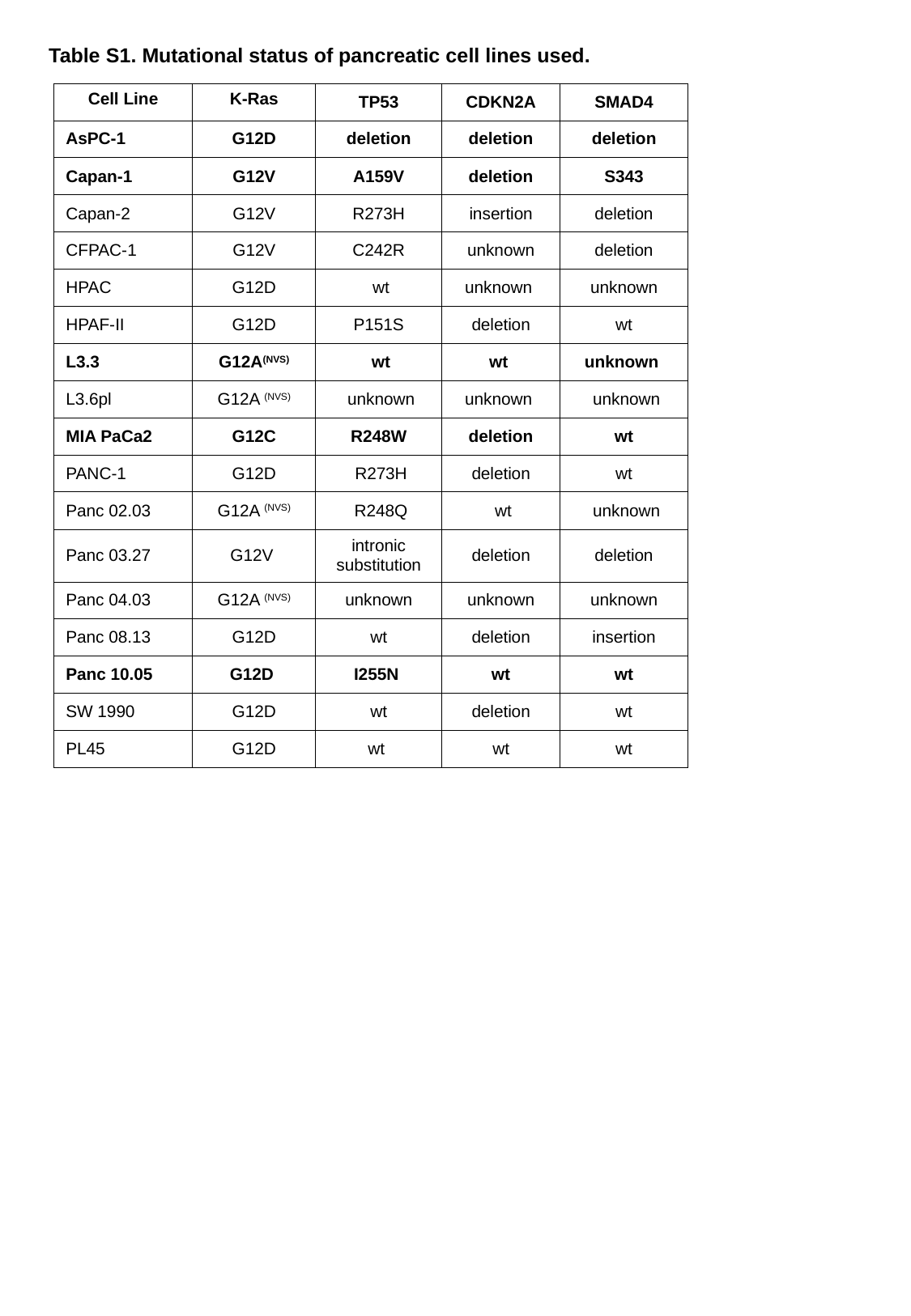

Table S1. Mutational status of pancreatic cell lines used.
| Cell Line | K-Ras | TP53 | CDKN2A | SMAD4 |
| --- | --- | --- | --- | --- |
| AsPC-1 | G12D | deletion | deletion | deletion |
| Capan-1 | G12V | A159V | deletion | S343 |
| Capan-2 | G12V | R273H | insertion | deletion |
| CFPAC-1 | G12V | C242R | unknown | deletion |
| HPAC | G12D | wt | unknown | unknown |
| HPAF-II | G12D | P151S | deletion | wt |
| L3.3 | G12A(NVS) | wt | wt | unknown |
| L3.6pl | G12A (NVS) | unknown | unknown | unknown |
| MIA PaCa2 | G12C | R248W | deletion | wt |
| PANC-1 | G12D | R273H | deletion | wt |
| Panc 02.03 | G12A (NVS) | R248Q | wt | unknown |
| Panc 03.27 | G12V | intronic substitution | deletion | deletion |
| Panc 04.03 | G12A (NVS) | unknown | unknown | unknown |
| Panc 08.13 | G12D | wt | deletion | insertion |
| Panc 10.05 | G12D | I255N | wt | wt |
| SW 1990 | G12D | wt | deletion | wt |
| PL45 | G12D | wt | wt | wt |
