# Supplementary material for: K-RAS Mutant Pancreatic Tumors Show Higher Sensitivity to MEK than to PI3K Inhibition In Vivo
Source: PLoS One. 2012 Aug 31;7(8):e44146. doi: 10.1371/journal.pone.0044146 (PMC3432074; doi:10.1371/journal.pone.0044146)
Supplement: Table S2 — Antitumor activities obtained in pancreatic models upon K-RAS knock down. Data shown in Figure 2B and 2C was analyzed by calculating the doubling time of the tumors, T/C (treatment/control) on day 18 (minimum treatment period), T/C on the last day of each study, and Δ tumor volume between the start and end of the study, as well as the area under the curve (AUC). Statistics were calculated by performing a t-test. No t-test calculation was possible for the doubling time of the model Capan-1 K-RAS sh236, as there was no tumor growth for 2 tumors in the doxycycline treated group resulting in infinite doubling times. An outlier determined by the Grubb’s test amongst the doubling times calculated for the model AsPC-1 K-RAS sh236 was not considered in the analysis. (PPT) [file pone.0044146.s005.ppt]

## Slide 1
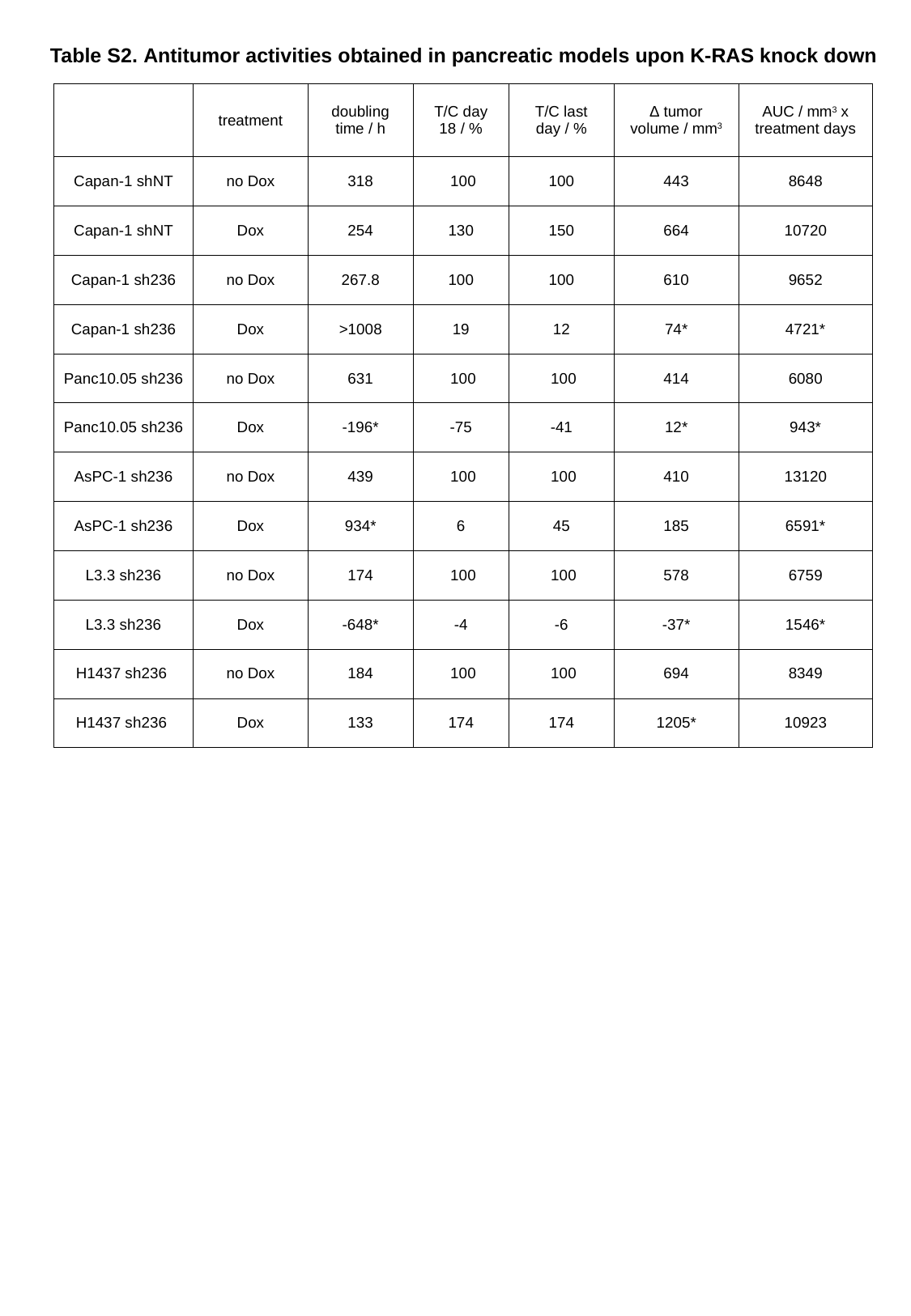

Table S2. Antitumor activities obtained in pancreatic models upon K-RAS knock down
| | treatment | doubling time / h | T/C day 18 / % | T/C last day / % | ∆ tumor volume / mm3 | AUC / mm3 x treatment days |
| --- | --- | --- | --- | --- | --- | --- |
| Capan-1 shNT | no Dox | 318 | 100 | 100 | 443 | 8648 |
| Capan-1 shNT | Dox | 254 | 130 | 150 | 664 | 10720 |
| Capan-1 sh236 | no Dox | 267.8 | 100 | 100 | 610 | 9652 |
| Capan-1 sh236 | Dox | >1008 | 19 | 12 | 74\* | 4721\* |
| Panc10.05 sh236 | no Dox | 631 | 100 | 100 | 414 | 6080 |
| Panc10.05 sh236 | Dox | -196\* | -75 | -41 | 12\* | 943\* |
| AsPC-1 sh236 | no Dox | 439 | 100 | 100 | 410 | 13120 |
| AsPC-1 sh236 | Dox | 934\* | 6 | 45 | 185 | 6591\* |
| L3.3 sh236 | no Dox | 174 | 100 | 100 | 578 | 6759 |
| L3.3 sh236 | Dox | -648\* | -4 | -6 | -37\* | 1546\* |
| H1437 sh236 | no Dox | 184 | 100 | 100 | 694 | 8349 |
| H1437 sh236 | Dox | 133 | 174 | 174 | 1205\* | 10923 |
